# The utility of CXCL13 and circulating CXCR5^+^ T cell detection in the diagnosis of systemic lupus erythematosus associated nephritis

**DOI:** 10.3389/fimmu.2025.1657350

**Published:** 2025-10-14

**Authors:** Yu Liu, Si-Yu Tan, Meng-Ke Huang, Yun-Long Yang, Lu Hui, Ting Liu

**Affiliations:** ^1^ Department of Laboratory Medicine, West China Second University Hospital, and West China Hospital, Sichuan University, Chengdu, China; ^2^ State Key Laboratory of Biotherapy and Cancer Center/National Collaborative Innovation Center for Biotherapy, Sichuan University, Chengdu, China; ^3^ Key Laboratory of Obstetric & Gynecologic and Pediatric Diseases and Birth Defects of Ministry of Education, Sichuan University, Chengdu, China; ^4^ Department of Clinical Laboratory, Honghui Hospital, Xi’an Jiaotong University, Xi’an, China

**Keywords:** systemic lupus erythematosus, lupus nephritis, CXCL13, CXCR5+ T cell, biomarker

## Abstract

**Introduction:**

CXCL13 regulates the homing of lymphocytes. It is highly expressed in kidney and serum of SLE patients, and correlates with disease activity, but its value in LN is unclear. This study was to investigate the expression and diagnostic value of CXCL13 and its regulated CXCR5^+^ T cells in LN.

**Methods:**

In mice, the association of serum CXCL13 levels with renal function was analyzed. And CXCR5^+^ T cell was detected by H&E staining and immunofluorescence in kidney, and by flow cytometry in spleen. In clinical studies, the association of CXCL13 and CXCR5^+^ T cells with disease activity was verified, and their diagnostic efficacy was assessed by ROC curves.

**Results:**

CXCL13 was elevated in both mouse and patients, and was positively correlated with disease severity. Serum CXCL13 had an AUC of 0.9287 in the diagnosis of SLE and 0.6244 in differential diagnosis of LN. And in MRL/lpr mice, CXCL13 was involved in the development of nephritis by promoting the recruitment of CXCR5^+^ T cells to the kidney. In the peripheral blood of LN patients, CXCR5^+^ T cells were significantly reduced. Combined detection of CXCR5^+^ T cell subsets resulted in an AUC of 0.9672 for the diagnosis of SLE and 0.7867 for the differential diagnosis of LN.

**Discussion:**

Serum CXCL13 has high accuracy in the diagnosis of SLE, and circulating CXCR5^+^ T cells may serve as a novel biomarker for the diagnosis of SLE and LN, which might complement the poor performance of serum CXCL13 in the diagnostic efficacy of LN.

## 1 Introduction

Systemic lupus erythematosus (SLE) is a complex autoimmune disease that affects multiple organs and tissues, including the skin, joints, kidneys, heart, lungs and central nervous system (CNS) (1). Lupus nephritis (LN) is one of the most common complications among SLE patients, impacting approximately 40-70% of SLE patients (2–4). Accumulated studies have shown that 10-30% of LN patients developing into end-stage renal disease (ESRD), significantly reducing the 10-year survival rate of SLE patients (5, 6). Moreover, previous studies have suggested that the pathogenesis of LN is closely associated with local deposition of autoantibodies and immune complexes in kidney, but there is growing agreement that infiltrating immune cells also contribute to kidney damage (7). In MRL/MpJ-Faslpr (MRL/lpr) mice, it was found that in the complete absence of circulating antibodies, these mice still developed nephritis characterized by T cells infiltration in the local inflammation of kidney (8). Clinical research have also confirmed the above view, finding that CXCR5^+^ T cells are enriched in inflamed kidneys and urine of patients with LN, and its expression is positively correlated with the disease activity index of SLE (9). These findings suggest that immune cells play a key role in the pathogenesis of SLE. Advancing our understanding of the role of immune cells in LN is expected to drive the development of novel treatment strategies to prevent nephritis, and hinder the progression to ESRD (10, 11).

C-X-C motif chemokine ligand 13 (CXCL13) is a kind of chemokine that regulates the homing of lymphocytes (12). It is widely involved in the pathogenesis of many autoimmune diseases and inflammatory diseases (13–15). Accumulated studies have shown that the serum expression of CXCL13 closely related to the disease activity and severity of SLE, and can be used as a potential biomarker of the disease (16). Additionally, CXCL13 and its corresponding receptor CXCR5 are also highly expressed in immunohistochemical staining sections of kidney tissues from SLE patients (17). In particular, specific neutralization of CXCL13 with anti-CXCL13 antibody can effectively decrease the Th17/Treg ratio in spleen, therefore alleviating kidney damage in MRL/lpr mice, which is a well-studied animal model for lupus and a generally accepted spontaneous model of LN (18, 19). These findings suggest that CXCL13 plays a critical role in the pathogenesis of SLE. However, less attention has been given to its effects on LN and also the regulation of CXCR5^+^ T cells during the disease process.

Therefore, in this study, we aimed to evaluate the expression of CXCL13, as well as the CXCR5^+^ T cells in the peripheral blood of LN patients and mice. Their correlation with disease activity was further investigated. Through this study, we may find a novel biomarker to meet the clinical need for better monitoring of LN.

## 2 Materials and methods

### 2.1 Study subjects

SLE patients (n =82) and healthy controls (HC, n = 38) were recruited from West China Hospital, Sichuan University. And we further subdivided the SLE patients into LN (n = 44) and non-LN (n = 38) groups. The diagnosis of SLE and LN was made by experienced clinicians in the rheumatology and immunology department based on accepted criteria set by the American College of Rheumatology (20, 21). This study was approved by the Medical Ethics Committee of West China Hospital, Sichuan University. Since the study used the remaining samples from clinical tests, no additional biological samples were collected, and the clinical treatment process of patients was not affected, the study was granted waivers of consent.

### 2.2 Biochemical and immunological tests

The concentration of serum complement C3 was determined by using an immunoturbidimetry commercial kit (Immage 800 analytical systems, Beckman Coulter Inc., USA). The biochemical indicators of renal function, including serum creatinine (Cr), urea nitrogen (BUN), serum albumin (Alb), and hemoglobin (Hb) were measured with Cobas C702 (Roche Diagnostics, Indianapolis, IN) and Sysmex XN-9100 (Sysmex, Kobe, Japan). The concentrations of CXCL13 were quantified by enzyme-linked immunosorbent assay (ELISA) according to the manufacturer’s protocols (Absin, Shanghai, China).

### 2.3 Animal experiments

Nine-week-old female MRL/lpr mice and C57BL/6 mice were purchased from Cavens Laboratory (Chang Zhou, China). All mice were housed under specific pathogen-free (SPF) conditions and sacrificed at 17 weeks of age (n=10/group). Spleens were collected, photographed and weighed, and then prepared into single-cell suspension for flow cytometry detection. Orbital blood was collected and performed for complete blood count (CBC) using auto hematology analyzer (BC-2800vet, Mindray Animal Care, China). The contents of serum Alb and Cr were determined by automatic biochemical analyzer (Chemray 240/800, Rayto Life and Analytical Sciences, China). The contents of serum CXCL13 was detected by commercial ELISA kit according to the instruction (R&D Systems). Furthermore, fresh 24 hours urine was collected at the end of the experiment day for urine Cr test (C011-2-1, Nanjing, China). All studies were approved and supervised by West China Second Hospital Animal Care and Use Committee, Sichuan University.

### 2.4 Histopathological analysis

For Hematoxylin and eosin (H&E) analysis, kidneys were fixed in 4% paraformaldehyde and embedded in paraffin, then sectioned at 5 μm. The lupus kidney damages were assessed by two pathologists using a previously reported grading method (22): 0 = normal; 1 = mild (cell proliferation and/or infiltration); 2 = moderate (membranoproliferation, lobulation, or hyaline deposition); 3 = severe (crescent formation or global hyalinosis). Glomerulonephritis was scored as the average of the scores derived from 50 glomeruli. To detect the CXCR5^+^ T cells expression levels in kidney, renal sections were blocked with 3% BSA. Thereafter, the sections were stained with primary antibodies as follows: rabbit anti-mouse CXCR5 antibody (ab254415), rabbit anti-mouse CD4 antibody (GB13064-2) and rabbit anti-mouse CD8 antibody (GB114196). Then, the sections were stained with secondary antibodies. All the antibodies were purchased from R&D Systems, abcam and servicebio technology.

### 2.5 Flow cytometry

Anti-Mouse antibodies (BD Biosciences, San Jose, CA) included: anti-CD3- APC, anti-CD4-FITC, anti-CD8-PE and anti-CXCR5- PerCP-Cy5.5. Anti-Human antibodies (BD Biosciences, San Jose, CA) included: anti-CD3-PerCP, anti-CD4-FITC, anti-CD8-PE and anti-CXCR5-BV421. Blood cells were co-incubated with antibodies, and then detected with BD FACS Canto II (BD Biosciences, CA, USA). The data was analyzed with Kaluza Flow Cytometry Analysis Software.

### 2.6 Data analysis and statistics

Sample size was calculated by Power and Sample Size software (Type I error rate = 0.05, Power = 0.9), determining that a sample size of 11 subjects per group, totaling 33 subjects, would be sufficient to detect a significant difference between the three groups. All data were expressed as mean ± SEM. For pairwise comparisons, the Shapiro-Wilk test was first employed to assess normality. Where data exhibited a normal distribution, the t-test was applied; for non-normally distributed data, the Mann-Whitney U test was used. For three-group comparisons, the *P*-values underwent Bonferroni correction. Linear regression was used to determine statistically significant correlations. Normality was assessed via the Shapiro-Wilk test. Pearson correlation analysis was applied to variables meeting normality criteria, while Spearman’s rank correlation analysis was applied to variables failing to meet normality criteria. Receiver operating characteristic (ROC) curve analysis as well as the area under the curve (AUC) were conducted to evaluate the diagnostic accuracy. The integrated approach for combined testing utilizes logistic regression via SPSS. *P* < 0.05 indicated that the difference was statistically significant.

## 3 Results

### 3.1 CXCL13 is involved in the regulation of renal function in mice

**Figure 1:**
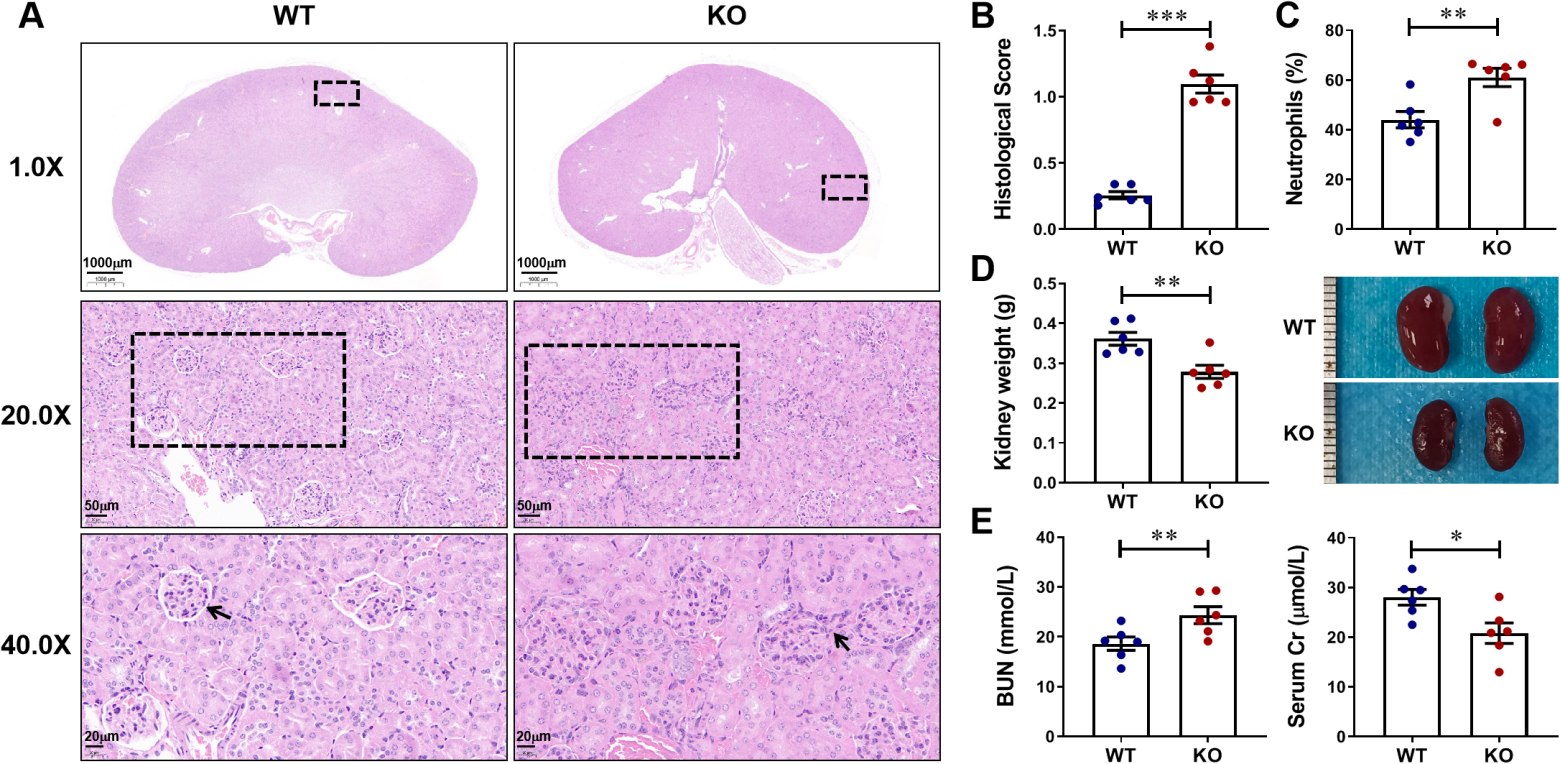
CXCL13 is involved in the regulation of renal function in mice. To investigate whether CXCL13 affects renal function, we naturally fed and observed CXCL13^-/-^ mice and age-matched WT mice. **(A–E)** At 34 weeks of age, CXCL13^-/-^ mice exhibited mild inflammation and kidney damage, including increased glomerular volume, inflammatory cell infiltration, elevated renal pathology scores in tissue sections, increased percentage of serum neutrophils, decreased kidney weight, decreased kidney size, elevated serum BUN, and reduced serum Cr. These experimental data suggest that CXCL13 deficiency leads to enhanced inflammatory response and decreased renal function in renal tissues of mice. Summary data are presented as mean ± SEM, *P < 0.05; **P < 0.01; ***P < 0.001.

Firstly, to investigate whether CXCL13 has an effect on the kidney, we naturally fed and observed CXCL13^-/-^ mice and age-matched wild-type (WT) mice. At 34 weeks of age, CXCL13^-/-^ mice showed significant renal pathological changes: increased glomerular size, blurred borders with inflammatory cell infiltration, and significantly elevated renal pathology scores (**Figures 1A, B**). This suggested that CXCL13 deficiency leads to kidney damage in mice. Significant elevation of the percentage of serum neutrophils in CXCL13^-/-^ knockout mice also suggested an inflammatory state of the kidney (**Figure 1C**). Gross renal lesions revealed that compared with WT mice, the kidney weights of CXCL13^-/-^ mice were reduced, suggesting structural disruption of the renal parenchyma (glomeruli and tubules) (**Figure 1D**). The results of renal function indices, including BUN and Cr, showed that CXCL13^-/-^ mice had significantly higher BUN and lower Cr, suggesting a decrease in glomerular filtration rate (GFR) (**Figure 1E**). These results suggest that CXCL13 deficiency leads to enhanced inflammatory response and decreased renal function in renal tissues of mice. That is, CXCL13 is involved in the regulation of renal function in mice.

### 3.2 Serum CXCL13 is elevated in MRL/lpr lupus mice and correlates with disease activity

**Figure 2:**
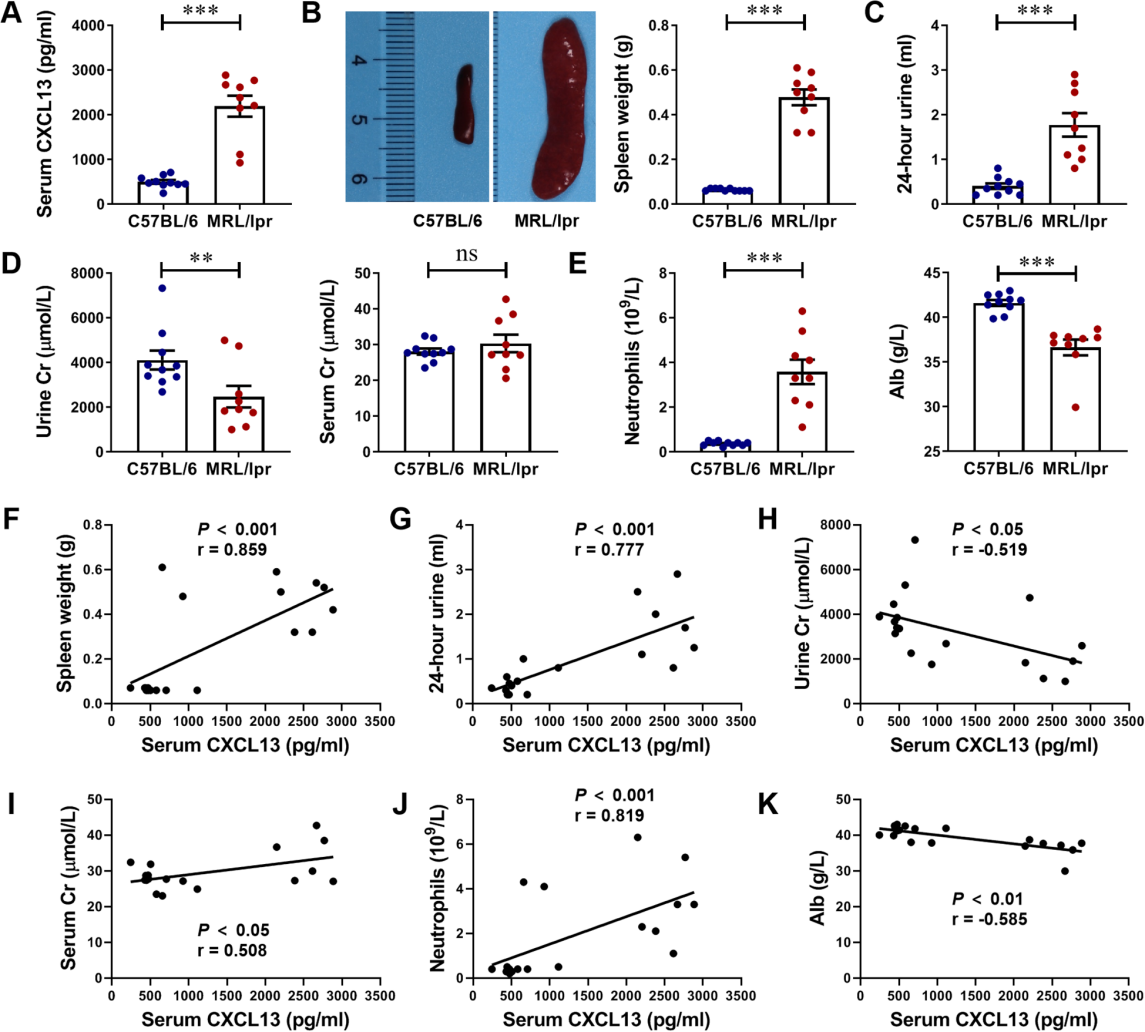
Serum CXCL13 is elevated in lupus-prone murine model and correlates with the clinical parameters of renal function. **(A)** ELISA analysis of CXCL13 in serum of MRL/lpr mice and C57BL/6 mice (n = 9-10/group). **(B–E)** MRL/lpr mice showed significant clinical manifestations of lupus nephritis at 17 weeks of age, including increased 24-hour urine volume, decreased urine Cr, increased serum Cr, increased neutrophils count, and decreased serum Alb levels. **(F–K)** Correlation between serum CXCL13 and spleen weight, 24-hour urine, urine Cr, serum Cr, neutrophils and Alb. These experimental data indicated that CXCL13 is significantly elevated in the serum of mice with lupus nephritis, and its expression is associated with kidney damage. Data are presented as mean ± SEM, *P < 0.05; **P < 0.01; ***P < 0.001.

Next, we successfully constructed an animal model of LN using MRL/lpr mice. MRL/lpr mice at 17 weeks of age showed significant clinical manifestations of LN, including splenomegalgia, increased 24-hour urine volume, decreased urine Cr, increased serum Cr, increased neutrophils, and decreased serum Alb levels (**Figures 2B-E**). Serum CXCL13 levels were detected by ELISA, and it was highly expressed in the serum of mice with LN (**Figure 2A**). Further analyze the correlation of serum CXCL13 with spleen weight, 24-hour urine volume, urine Cr, serum Cr, neutrophils count and serum Alb as indicators of disease severity in mice with LN. We found that: (1) serum CXCL13 was positively correlated with spleen weight, suggesting that its elevated levels could aggravate splenomegaly (**Figure 2F**). (2) Serum CXCL13 was positively correlated with 24-hour urine volume, suggesting that its elevated levels may exacerbate the damage of renal tubule interstitial function (**Figure 2G**). (3) Serum CXCL13 is closely related to Cr level, suggesting that its elevated levels may reduce urine Cr clearance rate, increase serum Cr retention, and aggravate the damage of glomerular filtration function (**Figures 2H, I**). (4) Serum CXCL13 was positively correlated with the level of neutrophils count, indicating that its elevated levels may exacerbate renal inflammatory response (**Figure 2J**). (5) Serum CXCL13 was negatively correlated with serum Alb levels, suggesting that its elevated levels may increase urinary protein loss caused by nephritis, and aggravate hypoproteinemia (**Figure 2K**). These results suggest that CXCL13 is significantly associated with disease severity in LN. There seems to be a contradiction between elevated serum CXCL13 and CXCL13 deficiency both leading to kidney damage in mice, which may be caused by the difference in the primary role of CXCL13 in the healthy and diseased states (15, 23–25). Thus, both deficiency and high expression of CXCL13 may lead to kidney damage.

### 3.3 The recruitment of CD4^+^CXCR5^+^ T cells at the site of glomerulonephritis in MRL/lpr lupus mice was significantly increased

**Figure 3:**
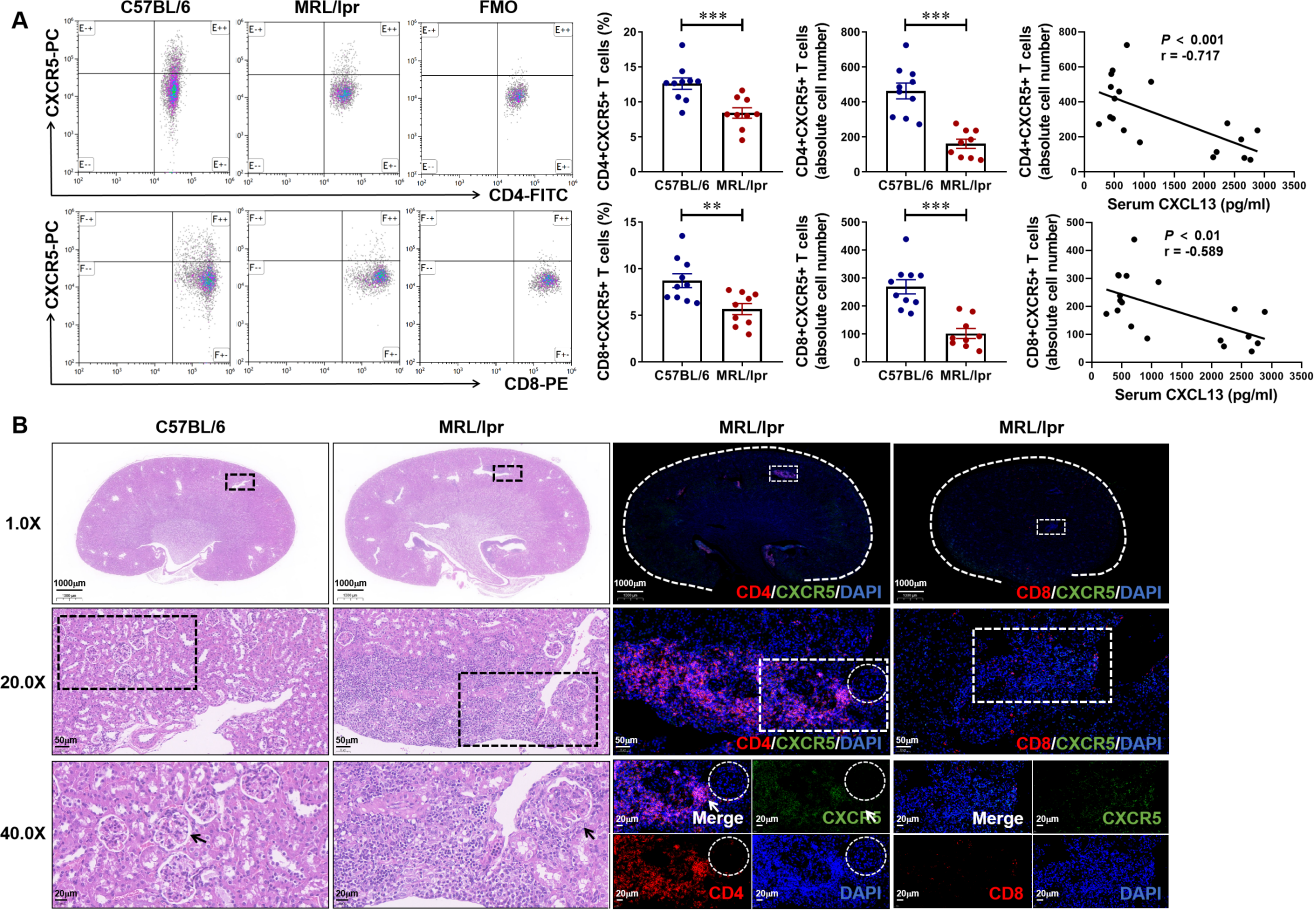
Abundance of CD4^+^CXCR5^+^ T cells are recruited at the kidney tissue inflammation site of mice with lupus nephritis. **(A)** Gated on CD3^+^ T cells. The percentage and absolute number of CD4^+^CXCR5^+^ and CD8^+^CXCR5^+^ T cells in MRL/lpr and C57BL/6 mice were analyzed (n = 9-10/group). The correlation between serum CXCL13 and the absolute number of CD4^+^CXCR5^+^ T cells and CD8^+^CXCR5^+^ T cells was further analyzed. **(B)** Representative H&E staining and immunofluorescence images of kidney tissue. Compared with CD8^+^ (Red) CXCR5^+^ (Green) T cells, CD4^+^ (Red) CXCR5^+^ (Green) T cells were more specific recruited at the inflammatory site of kidney tissue. Summary data are presented as mean ± SEM, statistically significant correlations were determined using linear regression. *P < 0.05; **P < 0.01; ***P < 0.001.

Flow cytometry analysis showed that the proportion and absolute number of CD4^+^CXCR5^+^ T cells and CD8^+^CXCR5^+^ T cells in the spleen of 17-week-old MRL/lpr mice with LN was significantly lower than that of C57BL/6 controls, and was negatively correlated with the expression level of serum CXCL13, that is, the higher the level of serum CXCL13, the fewer CD4^+^CXCR5^+^ T cells and CD8^+^CXCR5^+^ T cells in circulation (**Figure 3A**). For histopathology experiments, lupus-like renal disease in MRL/lpr mice is characterized by the development of glomerulonephritis and renal damage, including inflammatory cell infiltration, tubule atrophy, mesangial cell proliferation and interstitial fibrosis (**Figure 3B**). For immunofluorescence assay, we observed that CD4^+^CXCR5^+^ T cells were significantly recruited at the site of nephritis of LN mice (**Figure 3B**). These results indicate that CXCL13 regulates the migration of CD4^+^CXCR5^+^ T cells from the blood circulation to kidney tissues of mice with LN, and CD4^+^CXCR5^+^ T cells may play a key role in the occurrence and development of LN.

### 3.4 Serum CXCL13 is elevated in LN patients and correlates with clinical parameters

**Figure 4:**
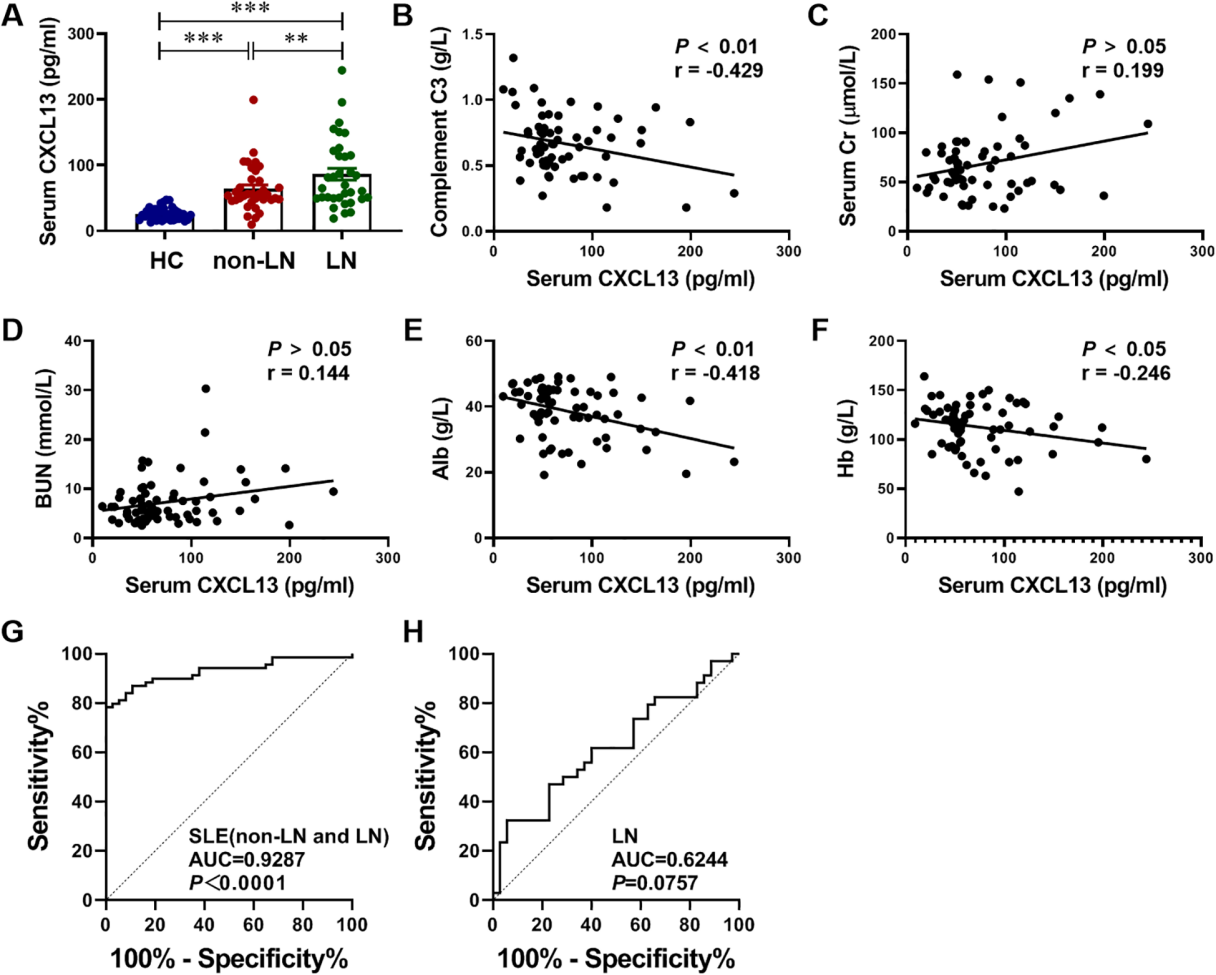
Serum CXCL13 expression is elevated in LN patients and correlated with disease severity. To identify differences in CXCL13 expression between HC and SLE patients, including LN patients and non-LN patients, **(A)** we performed ELISA tests on serum samples (n = 38/group), and further investigated its association with **(B)** disease severity biomarkers complement C3, **(C, D)** renal function related biomarkers Cr and BUN, **(E)** nutritional biomarker serum Alb, and **(F)** anemia biomarker Hb (n= 35-38/group). These experimental data indicated that serum CXCL13 was significantly elevated in LN patients, and its expression was positively correlated with disease severity. **(G)** ROC curve analysis of CXCL13 to distinguish SLE from HC. **(H)** ROC curve analysis of CXCL13 to distinguish LN from SLE. Summary data are presented as mean ± SEM, *P-adj < 0.05; **P-adj < 0.01; ***P-adj < 0.001.

To verify our finding in the clinical, we collected specimens from HC and SLE patients, including non-LN patients and LN patients. Firstly, serum CXCL13 levels were quantified by ELISA in 38 HC, 38 non-LN SLE patients and 38 LN patients. Notably, CXCL13 not only showed significant differences between HC and SLE patients (HC vs. non-LN: 26.03 ± 1.55 vs. 63.84 ± 5.97 pg/ml, *P* < 0.001; HC vs. LN: 26.03 ± 1.55 vs. 86.07 ± 9.02 pg/ml, *P* < 0.001), which also showed a significant difference between non-LN patients and LN patients (SLE vs. LN: 63.84 ± 5.97 vs. 86.07 ± 9.02 pg/ml, *P*-adj < 0.01) (**Figure 4A**). Additionally, the correlation of serum CXCL13 levels with disease severity biomarkers complement C3, renal function related biomarkers Cr and BUN, nutritional biomarker serum Alb, and anemia biomarker Hb was analyzed in patients with LN. Results demonstrated that serum CXCL13 levels exhibited statistically consistent and significant correlations with key clinical parameters of LN, including complement C3, serum Alb and Hb: (1) serum CXCL13 was negatively correlated with the level of complement C3, suggesting that its elevated levels may exacerbate complement depletion and aggravate disease severity (**Figure 4B**). (2) Serum CXCL13 was negatively correlated with Alb and Hb levels, indicating that its elevated levels may increase urinary protein and erythrocyte loss caused by nephritis, and exacerbate hypoproteinemia and anemia (**Figures 4E, F**). (3) Serum CXCL13 exhibited a positive correlation trend with serum Cr and BUN levels, though this did not attain statistical significance (**Figures 4C, D**). The relatively low correlation coefficients (r < 0.5) suggest that these associations are of limited strength and may be influenced by multiple factors, including patient heterogeneity and treatment regimens. However, these statistically significant trends consistently indicate that the elevated expression of CXCL13 in the serum of patients with LN has a certain correlation with disease severity. This also provides some support for the trends observed in animal experiments. We therefore further analyzed the diagnostic value of CXCL13 in SLE patients and its diagnostic value in differentiating LN patients. The results suggest that serum CXCL13 has high accuracy in the diagnosis of SLE (AUC = 0.9287), but the differential diagnostic value for LN needs to be improved (AUC = 0.6244) (**Figures 4G, H**). This may be due to the fact that serum CXCL13 originates from the release of elevated CXCL13 from inflammation of multiple organs into the bloodstream. Therefore, elevated serum CXCL13 may not be sensitive and specific enough in differentiating organ-specific diseases.

### 3.5 Clinical diagnostic value of CD4^+^CXCR5^+^ T cells combined with CD8^+^CXCR5^+^ T cells in patients with LN

**Figure 5:**
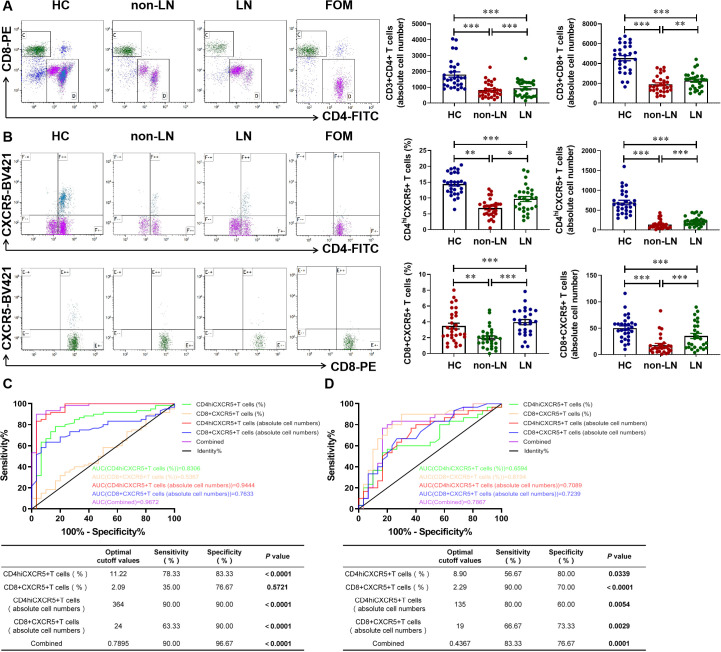
Clinical diagnostic value of CD4^+^CXCR5^+^ T cells combined with CD8^+^CXCR5^+^ T cells in patients with lupus nephritis. **(A)** Gated on CD45^+^CD3^+^ T cells. Comparison of CD4^+^ and CD8^+^ T cells numbers in HC and SLE patients including LN patients and non-LN patients (n = 29-30/group). **(B)** Gated on CD4^+^ CD8^+^ T cells. Comparison of CD4^+^CXCR5^+^ T cells and CD8^+^CXCR5^+^ T cells numbers in HC and SLE patients with or without nephritis (n = 28-30/group). **(C)** ROC curve analysis of different cell subsets to distinguish SLE from HC. **(D)** ROC curve analysis of different cell subsets to distinguish LN from SLE. These experimental data indicated that CD4^+^CXCR5^+^ and CD8^+^CXCR5^+^ T cells can diagnose SLE and serve as biomarkers to identify whether SLE patients have nephritis. *P-adj <0.05; **P-adj < 0.01; ***P-adj < 0.001.

In MRL/lpr mice, we noted that CD4^+^CXCR5^+^ T cells were significantly recruited at the nephritic site of renal tissues in LN mice. CXCR5^+^ T cells, as a CXCL13 regulated downstream indicator, may be valuable in the clinical diagnosis of LN patients. We measured the number of T cells and their CXCR5^+^ cell subpopulations in the blood cells of HC and SLE patients, both non-LN and LN patients, by flow cytometry. We found that CD4^+^ T cells and CD8^+^ T cells were significantly reduced in blood circulation in patients with SLE patients compared with HC, with a significant difference between non-LN patients and LN patients (**Figure 5A**). Interestingly, further experiments showed significant differences in the percentage and absolute number of CD4^+^CXCR5^+^ T cells and CD8^+^CXCR5^+^ T cells in the blood circulation of non-LN patients and LN patients, suggesting the potential of CXCR5^+^ T cells in differentiating patients with LN (**Figure 5B**). Reasonably, both the absolute cell number of CD4^+^CXCR5^+^ T cells and CD8^+^CXCR5^+^ T cells showed good diagnostic and differential diagnostic ability in patients. The AUC value for patients diagnosed with SLE was 0.9444 and 0.7633, respectively (**Figure 5C**), while the AUC value for the differentiation of LN patients was 0.7089 and 0.7239, respectively (**Figure 5D**). When combining these two cell subclusters as a diagnostic indicator, diagnostic efficacy was further enhanced (AUC = 0.9672 for SLE diagnosis and AUC = 0.7867 for differential diagnosis LN) (**Figures 5C, D**). These results suggest that CXCR5^+^ T cells may be a novel biomarker for the diagnosis of SLE. It is also promising to complement the poor performance of CXCL13 in the differential diagnosis of LN.

## 4 Discussion

LN belongs to the most serious complications of SLE and is still unsatisfactory in treatment (5, 6, 26). Early recognition is important in the management of LN (27). Currently, there are individual differences in the assessment of LN by conventional serological markers (urea, creatinine, urinary protein, etc.), and it is difficult to identify patients with asymptomatic LN. The chemokine CXCL13 is significantly overexpressed in the early stages of LN, and is one of the few inflammatory markers that are elevated before pathological changes in the kidney (28). We therefore expected to seek a serological assessment for early indication of kidney damage in SLE patients by evaluating the diagnostic efficacy of CXCL13 and its regulated CXCR5^+^ T cells in LN. In this study, we first found in wild-type and CXCL13^-/-^ knockout mice that CXCL13 deficiency resulted in decreased renal function and enhanced inflammatory response with infiltration of immune cells, suggesting that CXCL13 plays a crucial role in regulating renal function and maintaining renal immune homeostasis (**Figure 1**). Remarkably, in LN mice, we observed a significant elevation of serum CXCL13, with its expression levels positively correlated with disease severity and renal function impairment (**Figure 2**). The renal damage observed in both models—resulting from CXCL13 deficiency and from pathological overexpression of CXCL13—may appear contradictory. Actually, this reflects the characteristic biphasic effect of the biological function of CXCL13. Previous studies have confirmed that in the physiological conditions, CXCL13 is a key molecule in maintaining immune homeostasis, participating in the formation of secondary lymphoid organs and the maintenance of follicular architecture by regulating the migration and localization of B cells and Tfh cells (23). And its absence leads to abnormal follicular development, defective immune responses and autoimmune imbalances (23). Therefore, in the knockout experiments, aberrant development of secondary lymphoid organ follicular structures in CXCL13^-/-^ mice led to immune impairment, resulting in mildly enhanced inflammatory responses and decreased renal function in renal tissues. In pathological conditions such as lupus nephritis, ectopically overexpressed CXCL13 acts as a potent pro-inflammatory chemokine, regulating the migration of immune cells such as B lymphocytes and T lymphocytes to sites of inflammation. This exacerbates local inflammatory responses and tissue damage, thereby contributing to the onset and progression of the disease (15, 24, 25). In the LN mouse model, highly expressed CXCL13 regulates the migration of CD4^+^CXCR5^+^ T cells from the blood circulation to renal tissues in LN mice, thus playing a key role in the development of LN. This biphasic effect indicates illustrate that the normal expression of CXCL13 is important for maintaining the homeostasis of the immune system, and that both deficiency and high expression of CXCL13 may lead to kidney damage. This also suggests that CXCL13 neutralizing antibodies may be more clinically feasible than CXCL13 knockouts in terms of therapeutic strategies.

The association of serum CXCL13 with LN has also been validated in clinical studies (**Figure 4**). Animal experiments and clinical studies provide strong evidence for serum CXCL13 as a potential biomarker for LN. We therefore assessed the value of CXCL13 in differentiating SLE patients and LN patients by ROC curve. Our results confirmed that serum CXCL13 has considerable accuracy in the diagnosis of SLE, but limited efficacy in distinguishing LN (**Figure 4**). This is in accordance with previous findings of CXCL13 as a marker of renal involvement in SLE (AUC = 0.69 ± 0.058) (25). It may be possible to improve the diagnostic value by combining other indicators, such as plasmoblasts. Study shows that the percentage of plasmoblasts identifies renal involvement in SLE with an AUC of 0.795 (29). In addition, we considered that it is due to the elevation of serum CXCL13 in SLE patients results from diffusion of CXCL13 from inflammatory tissues of different organs into the circulation. Therefore, elevated serum CXCL13 may not be sensitive and specific enough in differentiating organ-specific diseases. Renal-derived CXCL13 may have a higher diagnostic value for differentiating renal involvement. This idea may find support in studies of cerebrospinal fluid (CSF) CXCL13 and neuroinflammation. CSF CXCL13 is highly specific in neuroinflammatory conditions, such as multiple sclerosis, and is little expressed in non-inflammatory states of the CNS (30, 31). Whereas serum CXCL13 levels may be elevated due to concurrent infections, even subclinical infections. Therefore, compared to serum CXCL13, CSF CXCL13 has diagnostic and prognostic value in neuroinflammation (30–35). Similarly, serum CXCL13 levels make it difficult to differentiate between SLE patients with renal involvement. Despite the current technical limitations in the detection of renal-derived CXCL13, CXCL13 as an early marker for LN is still worthy of in-depth study.

Notably, we identified a novel biomarker associated with CXCL13, circulating CXCR5^+^ T cells, which may be one of the important cells regulated downstream of CXCL13. Our study showed that CXCL13 regulates the migration of CD4^+^CXCR5^+^ T cells from the circulation to renal tissues of LN mice, thus participating in the development of LN (**Figure 3**). Consistent with the results detected in animal models, the proportion and absolute number of CXCR5^+^ T cells were significantly reduced in the serum of SLE patients (both non-LN patients and LN patients) (**Figures 5A, B**). Previous animal experiments and clinical studies on SLE have also found T-cell infiltration at sites of renal inflammation (8, 9). Our study confirmed these observations and identified its possible mechanism, which is elevated CXCL13 recruits CXCR5^+^ T cells to sites of inflammatory infiltration. This mechanism has been similarly validated in other models of inflammation. In the spinal cord of EAE models, CXCR5^+^ T-cell infiltration was significantly reduced in CXCL13^-/-^ mice (36). In the CSF of neuroinflammatory patients, CXCL13 levels were positively correlated with the proportion of CXCR5^+^ lymphocytes, with the strongest correlation being for CXCR5^+^CD4^+^ T cells (37). Furthermore, the diagnostic value of two cell subclusters of CXCR5^+^ T cells, including CD4^+^CXCR5^+^ T cells and CD8^+^CXCR5^+^ T cells, when used as a combined diagnostic index, was as high in patients with SLE as it was in differentiating patients with LN (**Figures 5C, D**). We therefore conclude that circulating CXCR5^+^ T cells are more sensitive and specific in identifying patients with LN than serum CXCL13.In conclusion, our study suggests that serum CXCL13 has high accuracy in the diagnosis of SLE, but the detection needs to be improved in the differential diagnosis of LN. And, we propose for the first time that circulating CXCR5^+^ T cells, especially CD4^+^CXCR5^+^ T cells, may be a novel biomarker for both diagnosing SLE and differentiating LN. It may serve as a complement to improve the diagnostic efficacy of serum CXCL13 in LN.

Additionally, our study contains certain limitations. In the animal experimentation, we selected healthy C57BL/6 mice as controls for MRL/lpr mice. Although the C57BL/6 strain provides a widely used healthy benchmark for assessing systemic immune activation and renal injury, some observed differences may stem from genetic background variations. Consequently, in subsequent mechanistic studies, we will use MRL/MpJ mice from a homologous background as controls to elucidate the specific contribution of the lpr mutation to phenotype development. In the clinical research, the conclusions of this study remain at an exploratory stage. Firstly, owing to the relatively limited sample size and single-center origin, we were unable to establish an independent validation cohort, thereby constraining the model’s generalizability. While the association between circulating CXCR5^+^ T cells and renal involvement in LN is suggestive, its ultimate clinical translational value requires further validation through prospective, multicenter studies involving larger cohorts. Secondly, key clinical data such as disease duration, SLEDAI scores, and detailed medication regimens were missing for some patients within the system. These uncontrolled confounding factors may influence biomarker levels. In future prospective studies, we will systematically collect such clinical raw data and apply multivariate regression analysis to more accurately assess the independent diagnostic efficacy of target biomarkers. Based on the current correlation findings, our further approach will directly validate the mechanistic role of CXCR5 in renal T-cell homing and LN pathogenesis through CXCR5 functional experiments. This will provide experimental evidence for potential therapeutic strategies.

## Data Availability

The original contributions presented in the study are included in the article/**Supplementary Material**. Further inquiries can be directed to the corresponding author/s.
