# Supplementary figures and images for: The utility of CXCL13 and circulating CXCR5+ T cell detection in the diagnosis of systemic lupus erythematosus associated nephritis
Source: Front Immunol. 2025 Oct 14;16:1657350. doi: 10.3389/fimmu.2025.1657350 (PMC12558933; doi:10.3389/fimmu.2025.1657350)

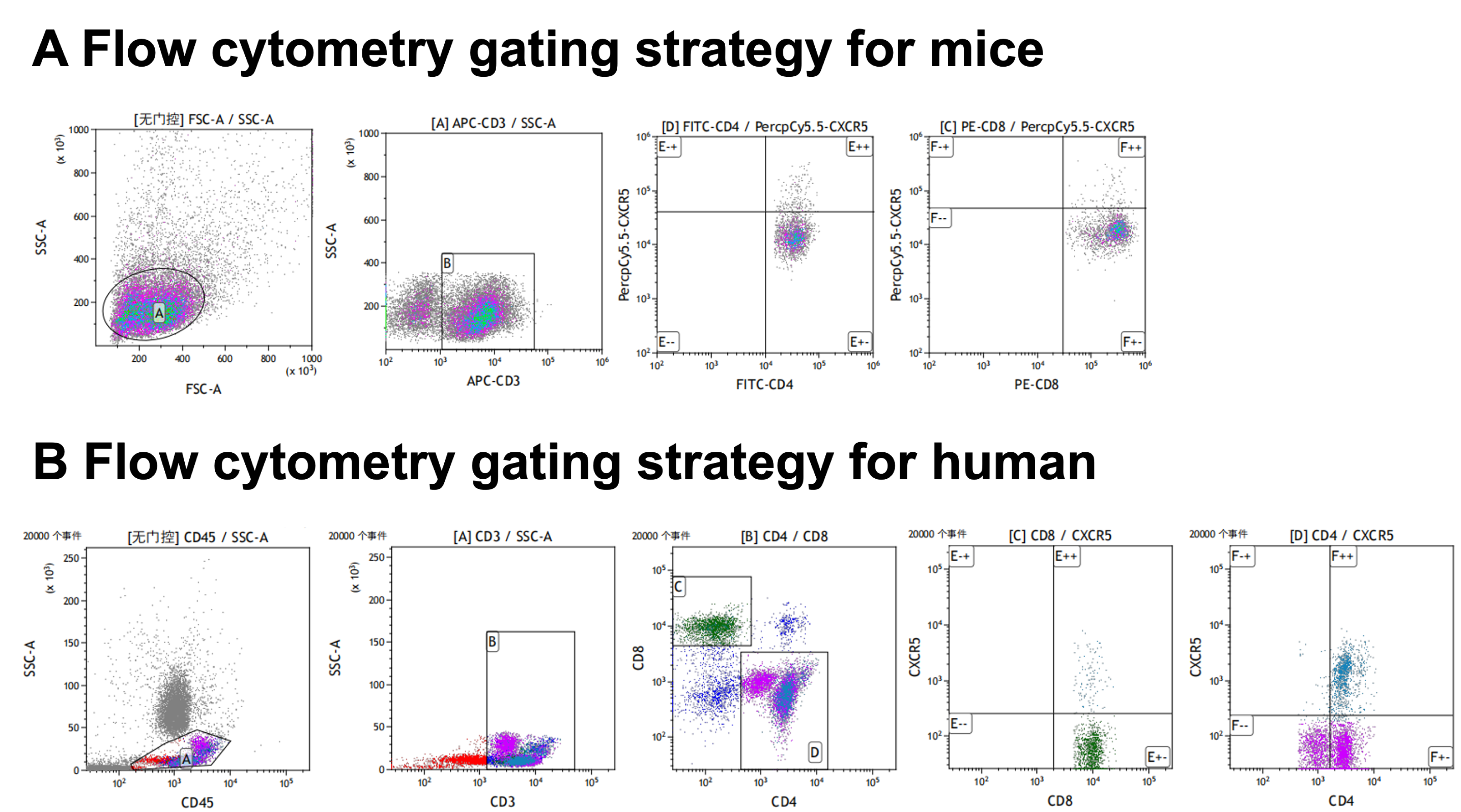

Supplement: Supplementary file 1 [file Image1.tif]
